# Supplementary figures and images for: Retinoic Acid-Mediated Inhibition of Mouse Coronavirus Replication Is Dependent on IRF3 and CaMKK
Source: Viruses. 2024 Jan 18;16(1):140. doi: 10.3390/v16010140 (PMC10819102; doi:10.3390/v16010140)

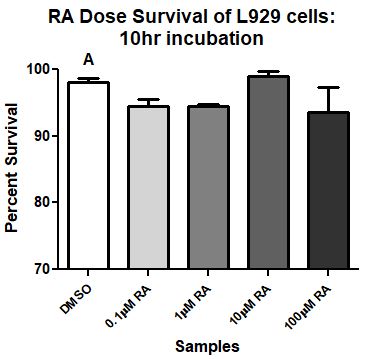

Supplement: Supplementary file 1 [file viruses-16-00140-s001.zip › Figure S1.JPG]

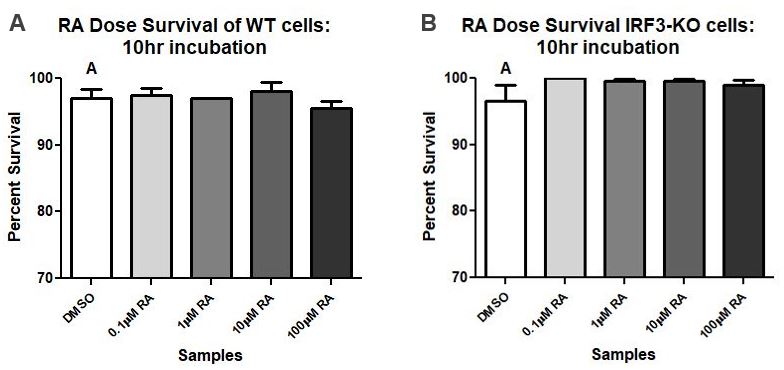

Supplement: Supplementary file 1 [file viruses-16-00140-s001.zip › Figure S2.JPG]
